# Spherical Aberration-Corrected Metalens for Polarization Multiplexed Imaging

**DOI:** 10.3390/nano11112774

**Published:** 2021-10-20

**Authors:** Shaodong Zhou, Kelei Xi, Songlin Zhuang, Qingqing Cheng

**Affiliations:** 1 Shanghai Key Laboratory of Modern Optical System, School of Optical-Electrical and Computer Engineering, University of Shanghai for Science and Technology, Shanghai 200093, China; ShaodongZ@outlook.com (S.Z.); 202310308@st.usst.edu.cn (K.X.); slzhuangx@aliyun.com (S.Z.); 2 State Key Laboratory of Terahertz and Millimeter Waves, City University of Hong Kong, Hong Kong, China

**Keywords:** terahertz, metasurface, metalens, spherical aberration

## Abstract

We present a terahertz spherical aberration-corrected metalens that uses the dynamic phase to achieve polarization multiplexed imaging. The designed metalens has polarization–dependent imaging efficiencies and polarization extinction ratios that exceed 50% and 10:1, respectively. Furthermore, opposite gradient phases can be applied to orthogonal polarizations to shift the imaging of the two polarized sources in the longitudinal and transverse directions. Indeed, we find that the metalens has a smaller depth-of-focus than a traditional metalens when imaging point sources with limited objective lengths. These results provide a new approach for achieving multifunctional beam steering, tomographic imaging and chiroptical detection.

## 1. Introduction

Large-aperture imaging lenses are used in a variety of high-resolution applications. Remote sensing and the Hubble telescope are among the best-known optical imaging system examples. When the aperture of imaging lens increases, spherical aberration becomes one of the main factors that restricts the resolution of the system. Spherical aberration can be eliminated by using combinations of special lenses such as ovoid singlet [1,2], liquid [3,4], and cylindrical lenses [5]. Aspheric lenses can be introduced to eliminate spherical aberration, but other aberrations appear alongside spherical aberration elimination to cause imaging distortion.

Metasurfaces, which are two-dimensional nanostructures, are composed of subwavelength scatterers and have strong potential for use in ultrathin device development. By changing the lateral geometrical parameters and the metasurface orientations, the geometric and dynamic phase of the metasurface can be used to modify the incident optical wavefront to the desired form. Capasso and coworkers constructed a “V”-shaped metasurface to verify the general Snell’s law and generated orbital-angular-momentum (OAM) beams [6]. Since then, OAM beams have been more efficiently generated in an integrated manner from metasurfaces [7,8,9,10]. Zhou and coworkers designed an “H”-shaped metasurface in which the phase profiles satisfy the gradient phase, thereby achieving the complete conversion of propagating waves to surface waves [11]. Huang et al. used a geometric phase to generate OAM beams in a wide frequency range [12]. Subsequently, metasurfaces have been widely used in the functional design of phases, amplitudes, and polarizations to achieve novel imaging functions such as holography [13,14,15,16], metalenses [17,18,19,20,21,22,23,24,25,26,27,28,29], and microscopy [30,31]. Some of the metasurface-based optical imaging provides advantages relevant to system integration [32,33] and smart programming [34] and many studies have reported the use of metasurfaces in endoscopes [35] and for beam shaping [36,37,38,39]. These features provide a method of eliminating spherical aberration in flat metalenses. For example, Capasso and coworkers proposed a metasurface designed on a curved surface to achieve aplanatic focusing [40,41]. Chen et al. designed an GaN aplanatic metasurface to achieve spectral tomographic imaging in the visible range [42]. Faraon and coworkers demonstrated a doublet metalens corrected over a wide range of incident angles [43]. In addition, the multiplexing of polarization carrying multiple functions has gradually become a cutting-edge research direction that contributes to techniques such as holography [14], anomalous beam steering [44,45,46,47,48], chiroptical-properties detection [49,50], and dual-band antennas [51]. Polarization multiplexing makes metasurface devices more powerful, functional, and compact. However, few studies have considered polarization multiplexing for spherical aberration-corrected designs. In practice, it is inevitable that the elimination of spherical aberration will pose a problem in the terahertz (THz) regime.

In this article, we simulated a polarization-multiplexed transmissive dielectric metalens that eliminates spherical aberration in the THz regime. In contrast with previous research on metalenses, we start with two arbitrary rays and go beyond the paraxial condition to obtain the spherical aberration-corrected focus phase profile. We show that the spherical aberration-corrected metalens images a point source located at a limited object length, and its full width at half maximum (FWHM) is 2.24mm in the longitudinal direction. In contrast, the FWHM of a conventional metalens is 5.92mm. Therefore, the proposed metalens has a greater depth of focus than the traditional metalens. Furthermore, upon applying different focal phases and opposite linear-gradient phases to the two orthogonal polarizations, the focal spots can be separated in the longitudinal and transverse directions. This work provides ideas for the design of multifunctional integrated metalenses, and should thus promote the application of metasurfaces in multifunctional beam steering, tomographic imaging and chiroptical detection.

## 2. Designs and Theory

**Figure 1:**
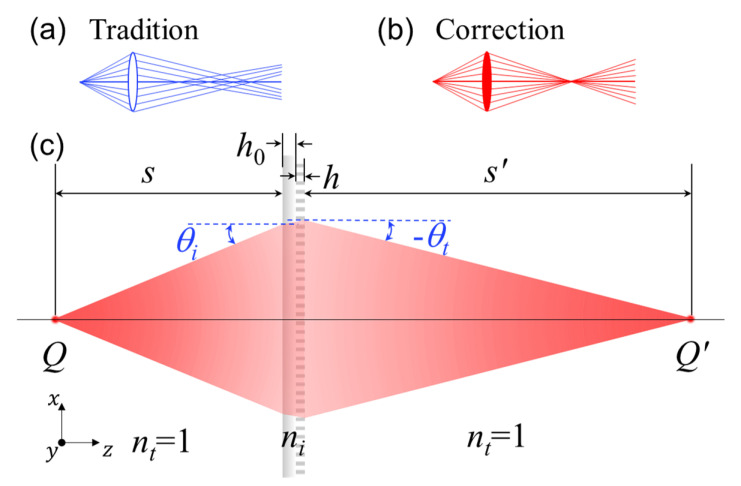
Schematic of spherical aberration-corrected metalens. $h_{0}$ and *h* are the height of the substrate and pillar in the metalens, respectively; *s* and $s^{\prime }$ are the object and image length, respectively; *Q* and $Q^{\prime }$ are the corresponding object and image positions; and $\theta_{i}$ and $\theta_{t}$ are the angles of incidence and of refraction, respectively. (**a**) For traditional spherical lenses, rays emitted from the source do not necessarily converge to the same point; (**b**) spherical aberration-corrected lens; and (**c**) spherical aberration-corrected metalens.

Spherical aberration occurs because spherical surfaces are not the ideal shape for a lens, but are by far the simplest shape to which glass can be ground and polished. As a result, spherical surfaces are often used, as shown in Figure 1a. To simplify the problem and illustrate spherical aberration, neglecting the thickness $h_{0}+h$ of the metalens in Figure 1c, light rays incident at all angles obey the generalized Snell’s law of refraction [6]:

(1) $$\frac{d\varphi (\rho )}{d\rho }=\frac{2\pi }{\lambda }\left(\sin\theta_{t}-\sin\theta_{i}\right),$$

where λ is the wavelength of the incident light, the distance ρ = ${\left(x^{2}+y^{2}\right)}^{1/2}$ and $\varphi \left(\rho \right)$ is the required phase profile at the position ρ in the radial dimension. For a traditional lens, the light rays projected from infinity (i.e., $\theta_{i}=0$) converge to the focal position, which is $s^{\prime }=f$ along the optical axis. Therefore, the phase delay ϕ(ρ) between rays at position ρ and at the center in the radial dimension is:

(2) $$\phi \left(\rho \right)=\varphi \left(\rho \right)-\varphi \left(0\right)=\frac{2\pi }{\lambda }\left(f-\sqrt{\rho^{2}+f^{2}}\right),$$

where *f* is the focal length. The traditional lens introduces spherical aberration when the objective source is located at a limited length. To eliminate spherical aberration in this case, the integration of Equation (1) with the differential relations given by $\sin\theta_{i}=\frac{d}{d\rho }{\left(s^{2}+\rho^{2}\right)}^{1/2}$ and $\sin\theta_{t}=-\frac{d}{d\rho }{\left(s^{\prime 2}+\rho^{2}\right)}^{1/2}$, yields:

(3) $$\frac{2\pi }{\lambda }\sqrt{s^{2}+\rho^{2}}+\varphi \left(\rho \right)+\frac{2\pi }{\lambda }\sqrt{s^{\prime 2}+\rho^{2}}=\frac{2\pi }{\lambda }s+\varphi \left(0\right)+\frac{2\pi }{\lambda }s^{\prime }.$$

Specifically, the point source at *Q* on the optical axis projects rays passing through the metalens within the distance ρ in the radial direction to be imaged at point $Q^{\prime }$—as shown in Figure 1c. Equation (3) ensures that the rays from the same point source to the corresponding image point have equal optical paths. We thus obtain a spherical aberration-corrected phase profile as a function of the working distance:

(4) $$\phi \left(\rho \right)=\varphi \left(\rho \right)-\varphi \left(0\right)=\frac{2\pi }{\lambda }\left(-\sqrt{s^{2}+\rho^{2}}-\sqrt{s^{\prime 2}+\rho^{2}}+s+s^{\prime }\right),$$

which is also derived from the generalized law of refraction (Equation (1)). By substituting $s^{\prime }$ into Equation (4) and using the Gaussian imaging formation $\left(s^{\prime }=\frac{sf}{s-f}\right)$, we obtain the same Equation (5) [42]:

(5) $$\phi \left(\rho \right)=\frac{2\pi s}{\lambda (f-s)}\left(-\frac{\left(f-s\right)\sqrt{s^{2}+\rho^{2}}}{s}+\frac{\sqrt{\left[2f^{2}-2fs+s^{2}+s\left(2f-s\right)\left(\frac{2s^{2}}{s^{2}+\rho^{2}}-1\right)\right]\frac{s^{2}+\rho^{2}}{s^{2}}}}{\sqrt{2}}-s\right).$$

Detailed specifications are provided in the Supplementary Materials.

Here, we choose the simple case *s* = 2f to capture the longitudinal images as clearly as possible. Based on Equation (5), the spherical aberration-corrected phase profile is:

(6) $$\phi \left(\rho \right)=\frac{4\pi }{\lambda }\left(2f-\sqrt{\rho^{2}+4f^{2}}\right).$$

This phenomenon is clearly shown in the Zemax simulation ray traces shown in Figure 1b, in which light rays from all incident angles converge to the same point when using the spherical aberration-corrected metalens. We can clearly find that the spherical aberration-corrected focal phase profile (in Equation (6)) is clearly different from the traditional focal phase as $\frac{2\pi }{\lambda }\left(f-\sqrt{\rho^{2}+f^{2}}\right)$. Explicitly, the design eliminates spherical aberration only for the chosen object length, so using a different objective length weakens the correction of the spherical aberration.

**Figure 2:**
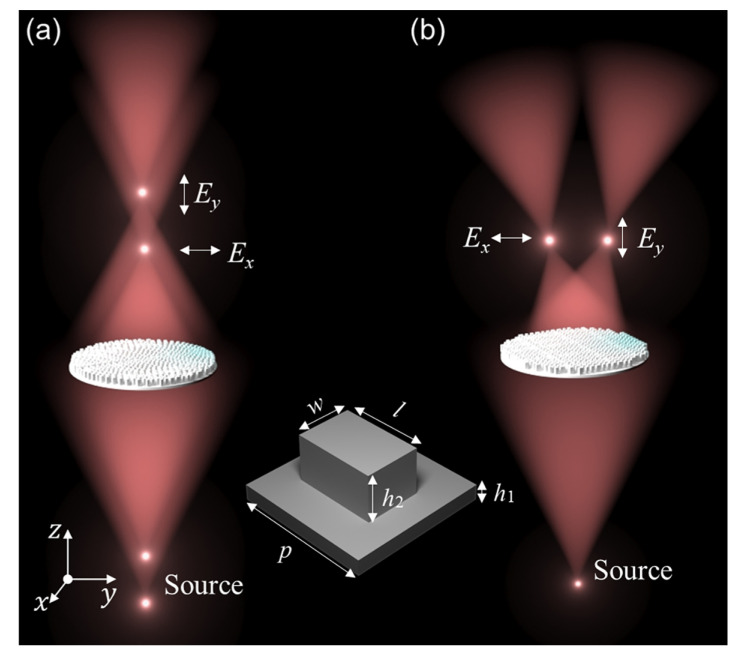
Schematic of spherical aberration–corrected metalens, with structural parameters shown: (**a**) two orthogonal polarization point sources at different object distances are imaged at different longitudinal positions; and (**b**) two orthogonal-polarization point sources at the same position are imaged at different transverse positions. The illustration shows the parameters of the rectangular unit cell.

To clearly present polarization multiplexing, consider two orthogonal-polarization point sources that produce spherical waves placed at different positions (Figure 2a) and at the same position (Figure 2b). In the former case, the image positions for the two polarizations are shifted longitudinally with respect to each other by the spherical aberration-corrected metalens. In the latter case, the metalens not only adds a constant-gradient phase in the *x* direction but also shifts the two polarized focal points apart in the transverse direction. Figure 2 also schematically illustrates how the selected rectangular silicon pillars are arranged on the silicon substrate ($n_{\mathrm{Si}}$ = 3.45).

## 3. Simulation Results

**Figure 3:**
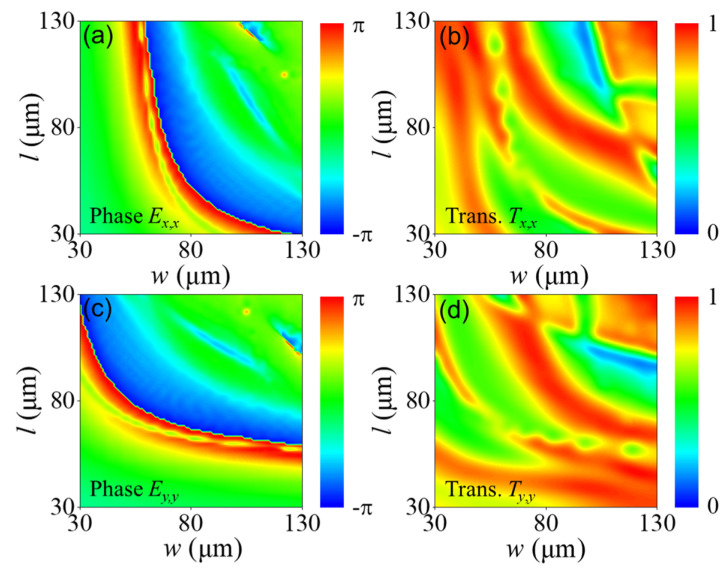
Polarization–dependent phase shift and transmission, which are symmetrical: (**a**,**b**) phase shift $E_{x,x}$ and transmission $T_{x,x}$, respectively, of the silicon pillar as functions of *w* and *l* under *x*-polarized incidence at 0.9 THz; (**c**,**d**) same as panels (**a**,**b**) but for *y*-polarized incidence.

We first established a structural database of the respective *x*- and *y*-polarized incidences using the commercially available software Lumerical FDTD. We gradually varied the pillar side lengths *w* and *l* from 30 to 130 μm in 2.5 μm steps. We fixed the substrate height to $h_{0}$ = 200 μm, the pillar height to *h* = 200 μm and the period to *p* = 150 μm. Here, we focused on controlling the THz wavefront at 0.9 THz. Considering the unit structure’s two-fold (C2) rotational symmetry [52,53,54], the dynamic phase $E_{y,y}$ and the amplitude response $T_{y,y}$ can be obtained by exchanging the *x* and *y* axes in the inset of Figure 2, which corresponds to: $\left(\begin{array}{c} E_{t}^{x}\\ E_{t}^{y} \end{array}\right)=\left(\begin{array}{cc} \left|T_{x,x}\right|\cdot E_{x,x} & \left|T_{x,y}\right|\cdot E_{x,y}\\ \left|T_{y,x}\right|\cdot E_{y,x} & \left|T_{y,y}\right|\cdot E_{y,y} \end{array}\right)\left(\begin{array}{c} E_{i}^{x}\\ E_{i}^{y} \end{array}\right)$ [55]. Each rectangular element must be designed to simultaneously satisfy the phase requirements of the spherical aberration-corrected metalens. The rectangular elements with two-fold (C2) rotational symmetry allows for the separate control of the transmission of the incident *x*- and *y*-polarized light without being influenced by cross-polarization coupling (i.e., $T_{x,y}$ and $T_{y,x}\approx 0$ ). Therefore, Figure 3a,c simply shows the calculated dynamic phase $E_{x,x}$ and $E_{y,y}$ as a functions of the pillar size (l,w), where the first subscript *x* or *y* denotes the incident polarization and the second subscript denotes the detected polarization. Figure 3b,d shows the corresponding transmission amplitudes $|T_{x,x}|$ and $|T_{y,y}|$. The simulated transmission coefficient indicates transmission from the silicon substrate through the pillar to the air for *x* or *y* incident polarization. These results form a structural database for use in the development of our design. The transmission amplitude, which represents the square root of the transmittance intensity, is expressed as $\left|T_{x,y}\right|=\frac{\left|T_{sx,y}\right|}{\sqrt{n_{s}}}$, where $T_{sx,y}$ is the simulated transmission coefficient.

**Figure 4:**
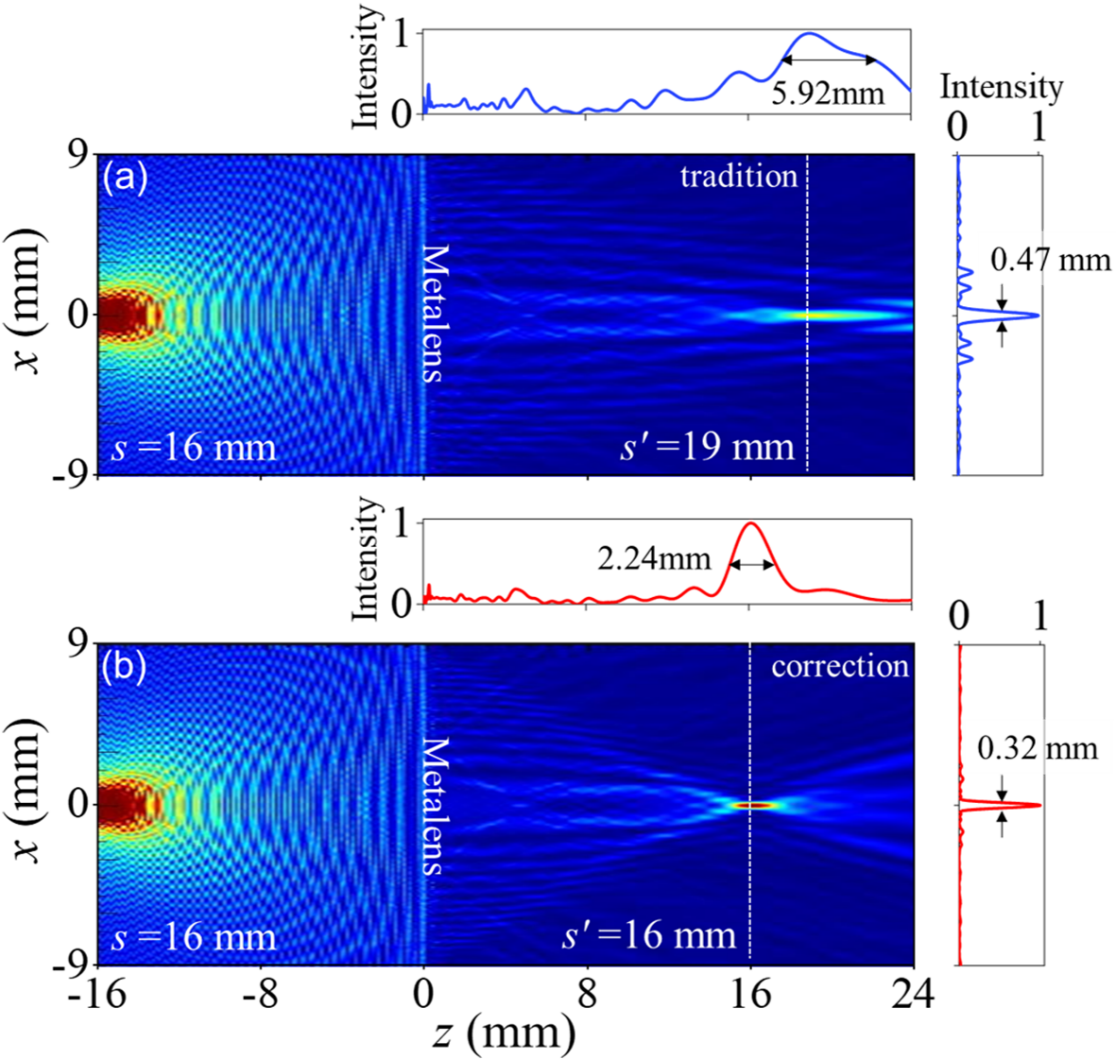
Traditional and spherical aberration–corrected metalens imaging. Simulation results for full-wave point-source imaging from (**a**) traditional and (**b**) spherical aberration-corrected metalenses. The insets show the corresponding ray-tracing results.

To confirm the theoretical analysis, full-wave simulations were performed using Lumerical FDTD. Figure 4a shows point imaging with a traditional metalens where the spatial phase profile follows Equation (2). There is a point source at s=16mm to the left and a point image at $s^{\prime }=19\;\mathrm{mm}$ to the right. The longitudinal and transverse FWHMs are 5.92 mm and 0.47 mm, respectively. However, for the spherical aberration-corrected metalens, the results are $s^{\prime }$ = 16, 2.24, and 0.32 mm, respectively, as shown in Figure 4b. The spherical aberration-corrected metalens greatly improves the focusing for a point source at an object distance of 2f, which indicates that high resolution can be achieved in tomographic imaging. In contrast, the traditional metalens produces an obvious divergence.

The versatile capabilities of the proposed polarization-multiplexed metalens allow it to separate the orthogonal polarization components in space to enable excellent polarization quality and high transmission. We thus propose a high-efficiency dual-focus lens with different focus lengths for *x* and *y* polarizations. Transverse and longitudinal polarization–dependent image splitting are led by the phase gradients in the respective directions [56,57] which can be expressed as

(7) $$\phi \left(x,y\right)=\frac{4\pi }{\lambda }\left(2f-\sqrt{{\left(x-x_{0}\right)}^{2}+y^{2}+4f^{2}}\right)+\frac{2\pi x}{\Lambda },$$

where Λ and $x_{0}$ are the period constant and the offset along the *x* direction, respectively. The first term in Equation (7) is the required phase for the focusing of light in the longitudinal direction. The second term is a constant-gradient phase in the *x* direction, which induces a polarization–dependent momentum shift (i.e., $\Delta k_{x}=-2\pi /\Lambda$) and generates transverse polarization–dependent splitting. The momentum-shift-driven motion of the real-space position of the *x* coordinate is determined by $x=-\Delta k_{x}z/k_{0}$, where $k_{0}$ is the wave vector in vacuum.

**Figure 5:**
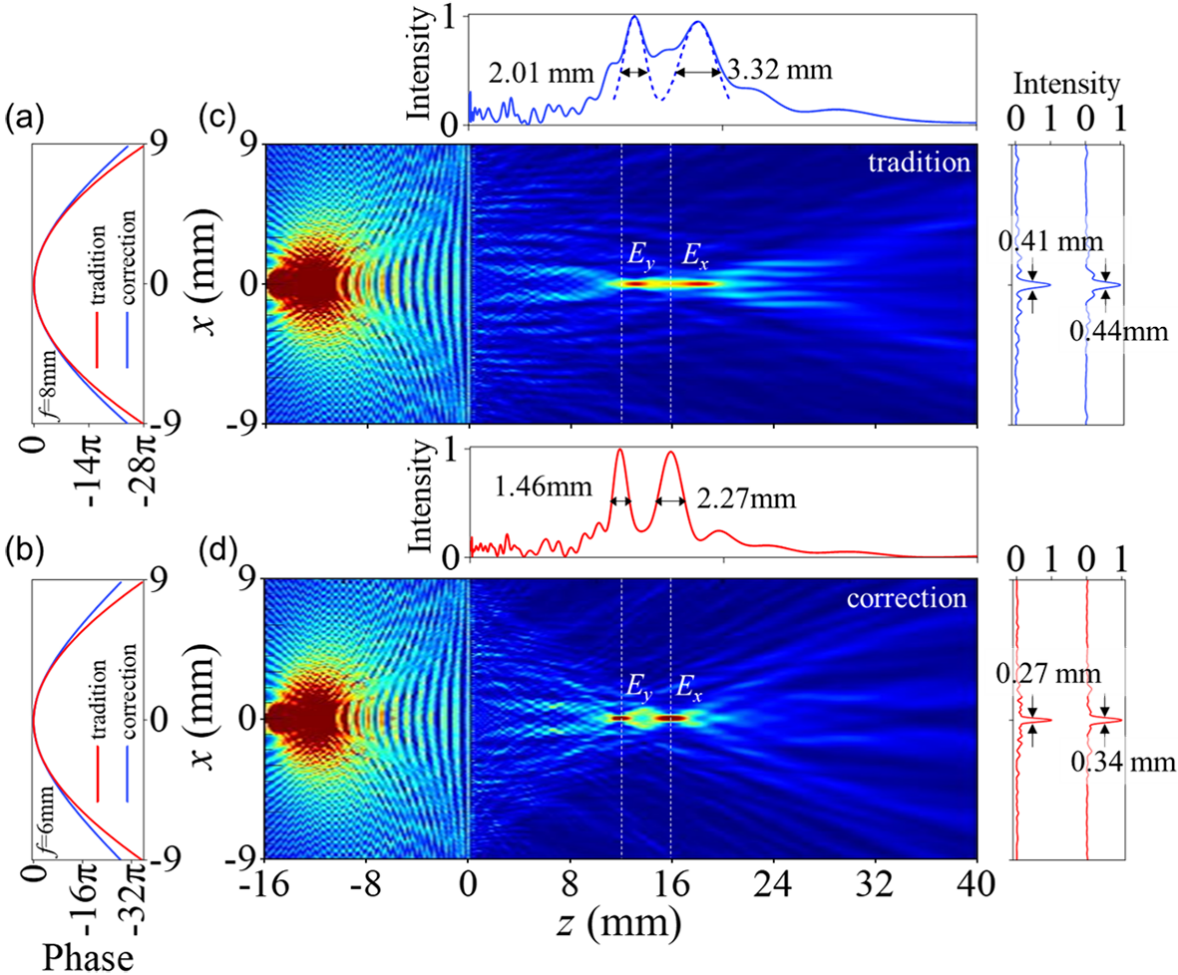
Polarization–dependent image separation in the longitudinal direction: (**a**) phase profiles of *x* polarization from traditional and spherical aberration-corrected metalenses with focal lengths of f=8mm; (**b**) phase profiles of *y*- polarization with focal lengths of f=6mm; (**c**) simulated distribution of electric-field intensity from polarization–dependent traditional imaging with image intensities in the transverse and longitudinal directions; (**d**) simulated distribution from polarization–dependent spherical aberration-corrected imaging.

First, we discuss longitudinal polarization–dependent splitting. Longitudinal polarization–dependent splitting is achieved via a longitudinal focusing effect. Thus, we consider only the first term in Equation (7), which influences the focusing position in the longitudinal direction. Figure 5a,b shows the phase profiles of traditional and spherical aberration-corrected metalenses, where the parameters are $f_{x}=8\;\mathrm{mm}$ for *x* polarization and $f_{y}=6\;\mathrm{mm}$ for *y* polarization at 0.9 THz. Figure 5c,d show the simulated electric-field distributions for polarizations multiplexing for the traditional and spherical aberration-corrected metalenses. In Figure 5c, the *x* and *y* polarization point sources are placed at $s_{x}=-16\;\mathrm{mm}$ and $s_{y}=-12\;\mathrm{mm}$, respectively. After imaging with the traditional metalens, the images are located at $s_{x}^{\prime }=19\mathrm{mm}$ and $s_{y}^{\prime }=13\;\mathrm{mm}$. The FWHM values of the *x* and *y* polarizations are ${\mathrm{FWHM}}_{x}=3.32\;\mathrm{mm}$ and ${\mathrm{FWHM}}_{y}=2.01\;\mathrm{mm}$ in the longitudinal direction (*z* axis), and ${\mathrm{FWHM}}_{x}=0.44\;\mathrm{mm}$ and FWHMy=0.41mm in the transverse direction (*x* axis). Compared with the imaging result produced using the spherical aberration-corrected metalens (Figure 5d), the images directly appear at $s_{x}^{\prime }=16\;\mathrm{mm}$ and $s_{y}^{\prime }=12\;\mathrm{mm}$. The corresponding FWHM values are ${\mathrm{FWHM}}_{x}=2.27\;\mathrm{mm}$ and ${\mathrm{FWHM}}_{y}=1.46\;\mathrm{mm}$ in the longitudinal direction, and ${\mathrm{FWHM}}_{x}=0.34\;\mathrm{mm}$ and ${\mathrm{FWHM}}_{y}=0.27\;\mathrm{mm}$ in the transverse direction. In terms of the precise focal length and smaller FWHM, the spherical aberration-corrected metalens produces a better image quality than the traditional metalens.

**Figure 6:**
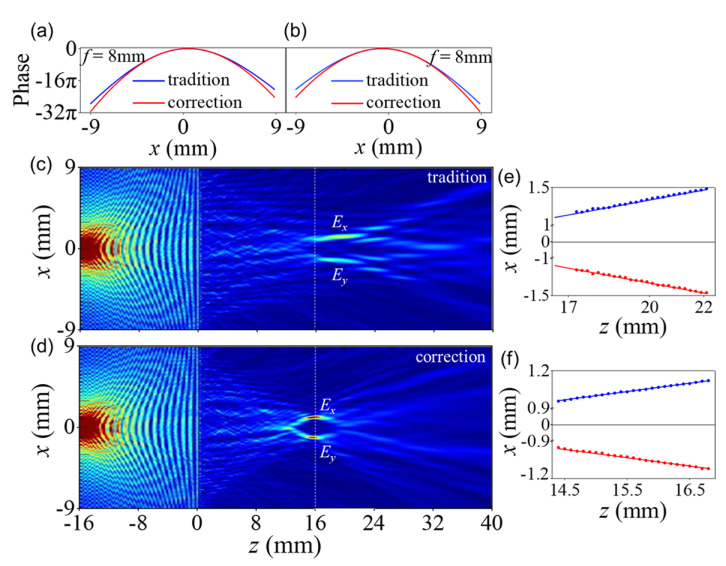
Separating polarization–dependent images in the transverse direction: (**a**) phase profiles of *x* polarization from traditional and spherical aberration-corrected metalenses with focal lengths of f=8mm, where the phase gradient is positive; (**b**) phase profiles of *y* polarization with focal lengths of f=8mm, where the phase gradient is negative; (**c**) simulated electric-field intensity of the traditional metalens with polarization–dependent imaging separation in the transverse direction; (**d**) simulated electric-field intensity of the spherical aberration-corrected metalens; and (**e**,**f**) intensity distribution fitting.

Then, to achieve horizontal spin-dependent splitting, we simultaneously considered the transverse gradient phase and horizontal focused phase. The period constants $\Lambda_{x}$ and $\Lambda_{y}$ are set to −5mm and 5mm, and the objective distance $s_{x}=s_{y}=16\mathrm{mm}$. The focus phase profiles are no longer symmetrical because of the gradient phases, as shown in Figure 6a for the *x* polarization and Figure 6b for the *y* polarization. Figure 6c shows the transverse offset of the traditional metalens from point-source imaging. For comparison, Figure 6d shows a result produced using a spherical aberration-corrected metalens. The polarized point sources are imaged on two spots that are closer to the theoretical imaging distance and have specific deflection values. The transverse shifts of the focal point grow as the propagation distance increases. Figure 6e,f show the transverse polarization–dependent real-space movements induced by the momentum shift during beam propagation. The solid lines are the theoretical values which are determined by using $x=-\Delta k_{x}z/k_{0}$. The results are x=−0.066z for the *x* polarization and x=0.066z for the *y* polarization. Figure 6e,f compare the results.

**Figure 7:**
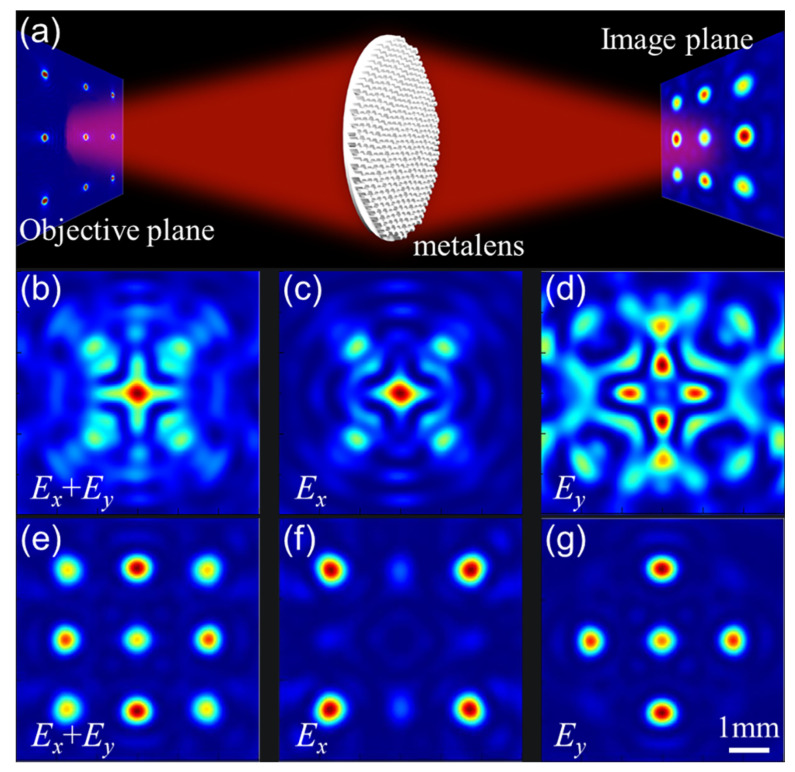
Dipole array consisting of *x* and *y* polarization at the object plane imaged using a spherical aberration-corrected metalens: (**a**) schematic of the optical measurement setup apparatus; the image plane for (**b**) the traditional and (**e**) the spherical aberration-corrected metalens contains an image of a 3×3 distributed point source; the *x*-polarized point sources for (**c**) the traditional and (**f**) the spherical aberration-corrected metalens are located at the four corners; the *y*-polarized point sources for (**d**) the traditional and (**g**) the spherical aberration-corrected metalens are located at the center of the square and at the center of each side, respectively.

Finally, we designed a spherical aberration-corrected metalens that implements polarizations multiplexing and high-polarization extinction for different parts of an image by simply arranging the polarizations. We constructed a 3×3 polarization point-source array in which the *x* and *y* polarization point sources are at the corners of a rectangle, the center of the rectangle, and the center of each side of a rectangle to illustrate the practical imaging quality of the spherical aberration-corrected metalens. The array is mounted at exactly s=2f=10mm in front of the metalens (radius ρ=3mm), which corresponds to a 4f optical configuration without image zoom, as shown in Figure 7a. Figure 7b,e show imaging without polarization. Furthermore, we calculate the polarization extinction ratios based on the *x* and *y* polarization imaging in Figure 7f and Figure 7g, respectively, to be 10:1 and 12.5:1, respectively. For a distinct comparison, the traditional metalens does not clearly show the imaging results of multiple point sources, as shown in Figure 7c,d. These simulation results confirm that the polarization–dependent multiplexed spherical aberration-corrected metalens provides strong extinction imaging capabilities. What we need to explain is that when the rectangular hole [58,59] in the metal film is on the order of the wavelength, the transmitted signal will act as the polarized point source. Therefore, it is completely feasible to use experiments to verify the imaging results.

## 4. Conclusions

In conclusion, we theoretically implemented a spherical aberration-corrected metalens that provides polarization–dependent multiplexed imaging in the terahertz regime. The spherical aberration-corrected metalens is composed of silicon pillars arranged on a square lattice. By altering the geometric parameters of the pillars, we obtain a family of structures that provides complete 2π phase coverage and a large working efficiency at the target frequency of 0.9 THz. The designed metalens offers high extinction and polarization multiplexing when applied to imaging. We expect that this technology will support further applications in terahertz photonics.

## Data Availability

The data presented in this study are available on request from the corresponding author.

## Supplementary Materials

The following are available online at https://www.mdpi.com/article/10.3390/nano11112774/s1, the derivation of Equation.
