# Supplementary material for: Spherical Aberration-Corrected Metalens for Polarization Multiplexed Imaging
Source: Nanomaterials (Basel). 2021 Oct 20;11(11):2774. doi: 10.3390/nano11112774 (PMC8624245; doi:10.3390/nano11112774)
Supplement: Supplementary file 1 [file nanomaterials-11-02774-s001.zip › nanomaterials-1345655-supplementary.pdf]

nanomaterials

MDPI

Supplementary Materials

Spherical Aberration-Corrected Metalens for Polarization Multiplexed Imaging

Shaodong Zhou [1], Kelei Xi [1], Songlin Zhuang [1] and Qingqing Cheng [1,2,*]

[1] Shanghai Key Laboratory of Modern Optical System, School of Optical-Electrical and Computer Engineering, University of Shanghai for Science and Technology, Shanghai 200093, China; ShaodongZ@outlook.com (S.Z.); 202310308@st.usst.edu.cn (K.X.); slzhuangx@aliyun.com (S.Z.)
[2] State Key Laboratory of Terahertz and Millimeter Waves, City University of Hong Kong, Hong Kong, China
* Correspondence: qqcheng@usst.edu.cn

Supplementary Materials for "the derivation of Equation (5)"

The generalized Snell's law of refraction: $\frac{d\varphi(\rho)}{d\rho}=\frac{2\pi}{\lambda}(\sin\theta_t-\sin\theta_i)$ show in Equation (1). The phase delay $\phi(\rho)$ between rays respectively at position ϱ and at the center in the radial dimension is: $\phi(\rho)=\varphi(\rho)-\varphi(0)=\frac{2\pi}{\lambda}\left(-\sqrt{s^2+\rho^2}-\sqrt{s'^2+\rho^2}+s+s'\right)$, as shown in Equation (4). By substituting s' into the upper equation and use the Gaussian imaging formation ($\frac{sf}{s-f}=s'$), the detailed specifications show as:

$$\phi(\rho)=\frac{2\pi}{\lambda}\left(-\sqrt{s^2+\rho^2}-\sqrt{\left(\frac{sf}{s-f}\right)^2+\rho^2}+s+\frac{sf}{s-f}\right)=\frac{2\pi}{\lambda}\left(-\sqrt{s^2+\rho^2}-\sqrt{\left(\frac{sf}{s-f}\right)^2+\rho^2}+\frac{s(s-f)+sf}{s-f}\right).$$

Deduction steps are given:

$$\Rightarrow\frac{2\pi s}{\lambda(f-s)}\left(\frac{(s-f)\sqrt{s^2+\rho^2}}{s}+\frac{s-f}{s}\sqrt{\left(\frac{sf}{s-f}\right)^2+\rho^2}-s\right)=\frac{2\pi s}{\lambda(f-s)}\left(\frac{(s-f)\sqrt{s^2+\rho^2}}{s}+\sqrt{\left(\frac{s-f}{s}\right)^2\left[\left(\frac{sf}{s-f}\right)^2+\rho^2\right]}-s\right).$$

A clear simplified form is: $\phi(\rho)=\frac{2\pi s}{\lambda(f-s)}\left(\frac{(s-f)\sqrt{s^2+\rho^2}}{s}+\sqrt{f^2+\frac{(s-f)^2}{s^2}\rho^2}-s\right)$.

We rewrite the expression in the second sqrt of the above formula to include only the numerator and denominator, as shown $\phi(\rho)=\frac{2\pi s}{\lambda(f-s)}\left(\frac{(s-f)\sqrt{s^2+\rho^2}}{s}+\sqrt{\frac{s^2f^2+s^2\rho^2-2sf\rho^2+f^2\rho^2}{s^2}}-s\right)$. To express the above formula more clearly, we extract the two parts of $s^2+\rho^2$ and $s^2+\rho^2$ from the second sqrt, as shown: $\phi(\rho)=\frac{2\pi s}{\lambda(f-s)}\left(\frac{(s-f)\sqrt{s^2+\rho^2}}{s}+\sqrt{\frac{s^2f^2+f^2\rho^2}{s^2}+\frac{(s^2\rho^2+s^4)+(s^2\rho^2-s^4)+(2s^3f-2sf\rho^2)+(-2s^3f-2sf\rho^2)}{2s^2}}-s\right)$. We next give the steps of the intermediate process from the above formula to Equation (5) in the main body.

$$\Rightarrow\frac{2\pi s}{\lambda(f-s)}\left(\frac{(s-f)\sqrt{s^2+\rho^2}}{s}+\sqrt{\frac{(s^2+\rho^2)(2f^2+s^2-2sf)}{2s^2}+\frac{(s^2-\rho^2)(2sf-s^2)}{2s^2}}-s\right)$$

$$\Rightarrow\frac{2\pi s}{\lambda(f-s)}\left(\frac{(s-f)\sqrt{s^2+\rho^2}}{s}+\frac{1}{\sqrt{2}}\sqrt{\left[2f^2+s^2-2sf+s(2f-s)\frac{s^2-\rho^2}{s^2+\rho^2}\rho^2\right]\frac{s^2+\rho^2}{s^2}}-s\right)$$

Finally, we intuitively derive the above formula to Equation (5).
